# Supplementary material for: Vulnerability Predictors of Post-Vaccine SARS-CoV-2 Infection and Disease—Empirical Evidence from a Large Population-Based Italian Platform
Source: Vaccines (Basel). 2022 May 26;10(6):845. doi: 10.3390/vaccines10060845 (PMC9230065; doi:10.3390/vaccines10060845)
Supplement: Supplementary file 1 [file vaccines-10-00845-s001.zip › vaccines-1709901-supplementary.pdf]

Supplementary Table s1. List of candidate conditions for predicting the risk of experiencing post-vaccine SARS-CoV-2 infection and severe fatal forms of COVID-19 infection

	#	Disease / condition	ICD-9 CM	ATC
Infectious and parasitic diseases	1	HIV infection	042.x, V08	J05AB14, J05AE, J05AF01, J05AF02, J05AF04, J05AF05, J05AF06, J05AF09. J05AG, J05AR, J05AX07, J05AX08, J05AX09, J05AX12
	2	Tuberculosis and Other infectious and parasitic diseases	010.x - 018.x, 001.x-009.x, 020.x-027.x, 030.x-0.41.x, 045.x–057.x, 060.x-066.x, 070.x-088.x, 090.x-104.x, 110.x-118.x, 120.x-139.x	J04AB
Neoplasms	3	Solid malignancies and Neoplasm of lymphatic and haematopoietic tissue	140.x-165.x, 170.x-176.x, 179.x-199.x, V58.0, 92.2, 200.x-208-x	L01, L03AC, L02BA01, L02BA02, L02BG02, L02BG03, L02BG04, L02BG06, L02BB01, L02BB03, L02AE02, L02AE04, L02AB01
	4	Benign neoplasm and carcinoma in situ	210.x-234.x	
Endocrine, nutritional and metabolic diseases, and immunity disorders	5	Hypothyroidism	243, 244.x	H03A, H03B
	6	Diabetes without insulin therapy	250.x, 348.0x, 357.2, 362.0, 366.41	A10B
	7	Insulin therapy		A10A
	8	Dyslipidaemia	272.2, 272.4	C10
	9	Obesity	278.0x	
	10	Weight loss	260-263.x	
	11	Disorders of fluid, electrolyte, and acid-base balance	276.x	
	12	Gout	274.x	M04AC01, M04AA, M04AB
	13	Other disorders of endocrine, nutritional and metabolic diseases	240.x-242.x, 245.x, 246.x, 249.x, 251.x, 252.8, 252.9, 253.x-259.x, 264.x-269.x, 270.x, 271.x, 272.0, 272.1, 272.3, 272.5-272.9, 273.x, 275.x, 277.x, 278.1-278.8 (except 277.0)	
	14	Disorders involving the immune mechanisms	279.x	
Diseases of the blood and blood-forming organs	15	Coagulation defects	286.x	B02B
	16	Autoimmune haemolytic anaemias, Other anaemias, Anaemias only tracked from drug therapy	280.x-282.x, 283.1-283.9, 284.x-285.x	B03A, B03B, B03XA01, L03AA

	17	Other diseases of the blood and blood-forming organs	287.x-289.x	
Mental disorders	18	Dementia / Alzheimer	290.0-290.4x, 331.0x	N06DA, N06DX01
	19	Psychosis	295.x, 297.x, 298.2-298.9, 299.1x	N05AD, N05AA, N05AB, N05AC, N05AX, N05AE, N05AF, N05AG N05AH, N05AL
	20	Depression	296.2, 296.3, 296.82, 298.0, 300.4, 301.12, 309.0x, 309.1x, 311.x	N06A
	21	Bipolar disorders	296.0x, 296.1x, 296.4x, 296.5x, 296.6x, 296.7x, 296.80, 296.81, 296.89, 296.9x, 298.1x	N05AN
	22	Alcohol abuse	291.1, 291.2, 291.5, 291.8x, 291.9, 303.9, 305.0x, V11.3x	N07BB01
	23	Drug addition	292.0x, 292.82-292.89, 292.9x, 304.x, 305.2x-305.9x	N07BB04
	24	Anxiety	300.0x	N05BA, N05BB01, N05CD, N05BC01, N05BC51, N05BX, N05CF, N05CX01, N06BX
	25	Other mental disorders	290.8, 290.9, 291.0, 291.3, 291.4, 292.1x, 292.2, 292.81, 293.x, 294.x, 299.0x, 299.8x, 299.9x, 300.0x-300.2x, 300.3, 300.5-300.9, 301.0, 301.10, 301.11, 301.2x-301.9x, 302.x, 303.x, 305.1, 306.x-308.x, 309.2x-309.4x, 310.x, 312.x-319.x	
Diseases of the nervous system and sense organs	26	Parkinson’s disease and parkinsonism	332.x	N04
	27	Multiple sclerosis	340	L03AB07, L03AB08, L04AA23, L04AA27, L03AX13, L04AA31, L04AA34, L03AB13, L04AX07
	28	Epilepsy and recurrent seizures	345.x	N03AF01, N03AB02, N03AA02, N03AA03, N03AA04, N03AE01, N03AD01, N03AG01, N05BA09, N03AG04, N03AX10, N03AG06, N03AF02, N03AX14, N03AX15
	29	Glaucoma	365.x	S01E
	30	Disorders of the eye and adnexa	360.x-379.x (except 365.x)	
	31	Diseases of the ear and mastoid process	380.x-389.x	
	32	Other diseases of the nervous system and sense organs	320.x-326.x, 330.x-331.x, 333.x-337.x, 340.x-344.x, 346.x-359.x	
	33	Ischaemic Heart Disease/Angina	410.x – 414	C01DA, C01DX

Diseases of the circulatory system	34	Heart failure	398.91, 402.11, 402.91, 404.11, 404.13, 404.91, 404.93, 428.x	
	35	Arrhythmia	426.10, 426.11, 426.13, 426.20-426.53, 426.60-426.89, 427.0, 427.2, 427.31, 427.60,427.9, 785.0x, V45.0x, V53.3x	C01BA, C01BC, C01BD
	36	Valvular diseases	093.20-093.24, 394.0x-397.1x, 424.00-424.91, 746.3x-746.6x, V42.2x, V43.3x	
	37	Vascular diseases	440.x, 441.2, 441.4, 441.7, 441.9, 443.1x-443.9x, 447.1, 557.1x, 557.9x, 785.4x, V43.4x	
	38	Cerebrovascular diseases	430.x-438.x	
	39	Hypertension	401.x-405.x	C03AA, C03AB, C03AH, C03AX01, C02CA04, C03BA02, C03BA03, C03BA04, C03BA05, C03BA07, C03BA08, C03BA09, C03BA10, C03BA11, C03DB01, C03DB02, C03EA, C09BA02, C09BA03, C09BA04, C09BA05, C09BA06, C09BA07, C09BA08, C09BA09, C09BB, C09DB, C09DA01, C09DA02, C09DA03, C09DA04, C09DA06, C09DA07, C09DA08, C02AB01, C02AB02, C02AC01, C02AC02, C02AC04, C02AC05, C02DB02, C02DB03, C02DB04, C02DC01, C02DD01, C02DG01, C02KA01, C02KB01, C02KC01, C02KD01, C02KX01, C09XA
	40	Coronary and peripheral vascular disease		B01AB, B01AX01, B01AD10, B01AD12, C04AD03, B01AC05
	41	Oral anticoagulant agents		B01AA, B01AE, B01AF
	42	Other diseases of the circulatory system	390.x-392.x, 393, 397.9, 398.90, 398.99, 411.8x, 412.x-417x, 420.x-423.x, 424.99, 425.x, 426.0, 426.12, 426.54, 426.9, 427.1, 427.32, 427.4x, 427.5, 427.61, 427.69, 427.8x, 429.x, 441.0x, 441.1, 441.3, 441.5, 441.6, 442.x,	

			443.0, 444.x-446.x, 447.0, 447.2-447.9, 448.x 451.x-459.x	
Diseases of the respiratory system	43	Chronic Obstructive Pulmonary Disease, Asthma, Chronic respiratory disease only tracked from drug therapy	490-492.x, 493.x, 494.x, 496	R03AA, R03AB, R03AC, R03DA, R03DB, R03DA20, R01AC01, R03BC01, R01AC51, S01GX01, S01GX51, R03BA
	44	Acute respiratory infections	460-466.x	
	45	Cystic Fibrosis	277.0	R05CB, R05FB01, R05FA01, A09AA02, R07AX02, R07AX30, R07AX31
	46	Other diseases of the respiratory system	470.x-478.x, 480.x-487.x, 495.x, 500.x-508.x, 510.x-519.x	
Diseases of the digestive system	47	Liver cirrhosis and other liver chronic diseases	571.x, 573.x	J05AP08, J05AP09, J05AP51, J05AP53, J05AP54, J05AP55, J05AP56, J05AP57, B05AA01
	48	Inflammatory bowel diseases (Ulcerative colitis and Chron’s disease)	555.x-556.x	A07EC01, A07EC02, A07EC03, A07EC04
	49	Chronic and acute pancreatitis	577.0-577.1	
	50	Other diseases of the digestive system	520.x-553.x, 557.x-570, 572.x, 574.x-576.x, 577.2-577.9, 578.x, 579.x	
Diseases of the genitourinary system	51	Chronic kidney disease	585, V45.1, V56.x, V03AE 580.x-584.x, 586, 587, 588.x-589.x	
	52	Other diseases of the genitourinary system	590.x-608.x, 610.x, 611.x, 614.x-629.x	
Diseases of the skin and subcutaneous tissues	53	Diseases of the skin and subcutaneous tissues, including No rheumatoid psoriasis	680.x-686.x, 690.x-695.x, 696.0, 696.2-696.5, 696.8, 697.x, 698.x, 700.x-709.x, 696.1	D05BB01, D05BB02, D05AX
Diseases of the musculoskeletal system and connective tissue	54	Autoimmune disease (Rheumatoid arthritis, Rheumatoid psoriasis, Anchylosing spondylitis, Systemic sclerosis, Systemic lupus erythematosus)	714.0, 696.0, 720.0, 710.1x, 710.0x	
	55	Other diseases of the musculoskeletal system and connective tissue	710.2-710.9, 711.x-713.x, 714.1x, 714.9x, 715.x-719.x, 720.1x-720.9x, 721.x-739.x	

Symptoms, signs and ill-defined conditions	56	Symptoms, signs and ill-defined conditions	780-799	
Other conditions	57	Transplantation	V42	L04AA01, L04AA02, L04AA03, L04AA04, L04AA05, L04AA06, L04AA08, L04AA09, L04AA10, L04AA11, L04AA12, L04AA14, L04AA15, L04AA16, L04AA17, L04AA18, L04AA19, L04AA21, L04AD01, L04AD02, L04AX01
	58	Chronic pain	338.2, 338.4	N02AA01, N02AG01, N02AE01, N02AB03, N02AA05, N02AA55, N02AA03, N02AX06
	59	Corticosteroids		H02
